# A single presumptive deworming may not suffice to reduce the burden of intestinal parasitic infections during pregnancy in rural Amhara, Ethiopia

**DOI:** 10.1186/s13104-025-07309-w

**Published:** 2025-06-03

**Authors:** Kalkidan Yibeltal, Firehiwot Workneh, Nebiyou Fasil, Estifanos Baye, Yunhee Kang, Workagegnhu Tarekegn Kidane, Sitota Tsegaye, Yoseph Yemane Berhane, Bethlehem Haymanot, Ingrid E. Olson, Mulatu Melese Derebe, Rose L. Molina, Blair J. Wylie, Grace J. Chan, Parul Christian, Alemayehu Worku, Anne CC Lee, Yemane Berhane

**Affiliations:** 1 Department of Reproductive Health and Population, Addis Continental Institute of Public Health, Addis Ababa, Ethiopia; 2 Department of Epidemiology and Biostatistics, Addis Continental Institute of Public Health, Addis Ababa, Ethiopia; 3 Department of Global Health and Health Policy, Addis Continental Institute of Public Health, Addis Ababa, Ethiopia; 4 Department of Pediatrics, Brigham and Women’s Hospital, Boston, MA USA; 5 Center for Human Nutrition, Department of International Health, Johns Hopkins Bloomberg School of Public Health, Baltimore, MD USA; 6 Department of Nutrition and Behavioral Science, Addis Continental Institute of Public Health, Addis Ababa, Ethiopia; 7 Addis Continental Institute of Public Health, Bahir Dar, Ethiopia; 8 Amhara Public Health Institute, Bahir Dar, Ethiopia; 9 Harvard Medical School, Boston, MA USA; 10 Department of Epidemiology, Harvard T.H. Chan School of Public Health, Boston, MA USA; 11 Department of Medical Critical Care, Boston Children’s Hospital, Harvard Medical School, Boston, MA USA; 12 Department of Obstetrics and Gynecology, Columbia University Irving Medical Center, New York, USA; 13 Department of Obstetrics and Gynecology, Beth Israel Deaconess Medical Center, Harvard Medical School, Boston, MA USA; 14 Department of Pediatrics, Warren Alpert Medical School of Brown University, Providence, RI USA

**Keywords:** Deworming, Intestinal parasite, Infection, Pregnancy, Ethiopia

## Abstract

**Objective:**

This study aimed to assess the prevalence of intestinal parasitic infections among pregnant women in the third trimester who received prior presumptive deworming in 12 health centers in the Amhara region, Ethiopia. This sub-study was part of the parent Enhancing Nutrition and Antenatal Infection Treatment (ENAT) study; a randomized clinical effectiveness study conducted to determine the effectiveness of packages of antenatal interventions to enhance maternal nutrition and infection management on birth outcomes.

**Results:**

Three hundred fifty women provided a stool sample in their 3rd trimester for screening using wet mount microscopy. All women had previously received 500 mg of presumptive mebendazole in the 2nd trimester. One in three women (109/350, 31.0%) were found to have a parasitic stool infection after prior deworming and 15% of women reported gastrointestinal symptoms. The most common infections were *Giardia lamblia (n =* 43, 37.4%), *Entamoeba histolytica (n =* 40, 34.8%), and *Hookworm (n =* 25, 21.7%). Six mothers had co-infections with at least two parasites with trophozoites of *Giardia lamblia* and *Entamoeba histolytica* co-infection being dominant.

## Introduction

Infections during pregnancy including intestinal parasitic infections (IPIs) may cause anemia and nutrient malabsorption and thereby contribute to poor pregnancy weight gain, fetal growth restriction, and adverse pregnancy outcomes like low birth weight and preterm birth [1–3]. Systematic reviews of studies in Ethiopia report an overall prevalence of intestinal parasitic infections among pregnant women of 29-31.8% [4, 5]. The major categories of intestinal parasites during pregnancy include soil-transmitted helminths and protozoa [5, 6]. In settings with a high geo-helminthic burden like Ethiopia, the World Health Organization recommends prophylactic deworming in either the second or third trimesters of pregnancy, to reduce the burden of hookworm and *Trichuris trichiura* infection [7–9]. However, there are no recommendations for protozoal intestinal infection prophylaxis.

In this sub-study, we aimed to assess the prevalence, patterns for intestinal parasitic infections among pregnant women in rural Amhara Ethiopia. Current Ethiopian antenatal care guidelines recommend presumptive deworming in the second trimester with mebendazole. Most studies conducted previously in Ethiopia were cross-sectional studies which assessed the prevalence of intestinal parasitic infections (IPIs) or coverage of deworming at a single time point [7, 10–13]. For studies, examine the prevalence of IPIs after provision of presumptive deworming. In the current study, we examine the prevalence of IPI’s among women in the third trimester of pregnancy post-prophylactic deworming.

The findings of this study provide evidence on the literature gap to guide future strategies on the control IPIs in pregnancy.

## Methods

This study is nested under the parent Enhancing Nutrition and Antenatal Infection Treatment (ENAT) study (ISRCTN15116516). ENAT study was 2 × 2 factorial pragmatic, open-label, randomized clinical effectiveness study conducted in rural health centers in the Amhara region, Northwest Ethiopia, to determine the effectiveness of packages of antenatal interventions to enhance maternal nutrition and infection management on birth outcomes. Further details about the ENAT study are published elsewhere [14]. ENAT participants who were randomized to the enhanced infection management package (EIMP) received routine presumptive deworming of a single dose of 500 mg mebendazole followed by wet-mount microscopy stool examination one month later with tailored treatment if they were positive. Treatment was guided per the National Ministry of Health treatment guidelines [15].

Stool samples were collected subsequently from a subset of 350 pregnant women consecutively enrolled in the EIMP arm presenting for antenatal care (ANC) in the 3rd trimester regardless of their symptoms and who had received a presumptive mebendazole (500 mg oral tablet) at least a month prior. Mothers were observed taking the mebendazole at the health center pharmacy. The stool samples were screened for intestinal parasites in the study health centers’ laboratory using the national standard of care, wet-mount microscopy [16, 17]. Wet mount microscopy has 37.1% sensitivity and 100% specificity in detecting intestinal parasites [18].

## Result

Post mebendazole deworming, 31% (109 cases out of 350 screened) of women had a parasitic stool infection in 3rd trimester, where 6 of them had coinfections with more than one parasite (Table 1). Of the 335 mothers provided data on the presence of gastrointestinal (GI) symptoms in the past 7 days (severe abdominal pain, diarrhea, nausea, vomiting, and constipation) on the day of stool sample collection, where only 50 (14.9%) had any one or more symptoms while reported having at least one symptom whilethe remaining 285 (85.1%) were asymptomatic. Among women who had parasitic stool infections in the 3rd trimester, approximately 19.4% reported GI symptoms (20/103). The three most common intestinal parasites identified in the study population were the protozoa, *Giardia lamblia* (*n* = 43/109, 39%), *Entamoeba histolytica* (*n* = 40/109, 37%), followed by *Hookworm* (*n* = 23/109, 21.1%). The detailed parasitic profile is indicated in Table 1.

**Table 1:** Prevalence and types of intestinal parasitic infections among pregnant women in 3rd trimester post-presumptive deworming (*n* = 350), Amhara region, Ethiopia

Parasites	Number (Percent)
Stool examinations completed	350 (100%)
Pregnant women with identified stool parasite(s)	109 (31.0%)
*Giardia lamblia* (Trophozoite & cyst)	43 (37.4%)
*Entamoeba histolytica* (Trophozoite & cyst)	40 (34.8%)
*Hookworm*	25 (21.7%)
*Ascaris lumbricoides*	3 (2.6%)
*Schistosoma mansoni*	1 (0.9%)
*Schistosoma haematobium*	1 (0.9%)
*Strongyloides stercoralis*	1 (0.9%)

The sociodemographic characteristics of the pregnant women in the study are shown in Table 2. The median maternal age was 25 (IQR 22, 30). The majority of mothers worked in agricultural labor and had no formal education. Most women reported having access to a public tap and dug well sources (*n* = 86.5%) for drinking water and improved toilet facilities (*n* = 230, 66.3%).

**Table 2:** Sociodemographic characteristics and correlations with intestinal parasitic infection (*n* = 350), Amhara region, Ethiopia

Variables	Variable categories	IPIs detected	IPIs not detected	Sig.
Age (years)	16–20	28 (36.8%)	48 (63.2%)	0.60
	21–25	30 (27.3%)	80 (72.7%)	
	26–30	35 (32.4%)	73 (67.6%)	
	31–35	9 (25.0%)	27 (75.0%)	
	36–45	7 (35.0%)	13 (65.0%)	
Marital status †	Never married/ never lived together	6 (46.2%)	7 (53.8%)	0.24
	Married/ living together	103 (30.8%)	232 (69.2%)	
Educational status ‡	No formal education	56 (37.3%)	94 (62.7%)	0.09
	Primary or lower	31 (29.3%)	75 (70.7%)	
	Secondary or higher	22 (24.2%)	69 (75.8%)	
Occupation †	Agriculture / daily laborer	63 (36.2%)	111 (63.8%)	0.12
	Informal	24 (24.5%)	74 (75.5%)	
	Waged occupation	22 (28.9%)	54 (71.1%)	
Family size ‡	<=2	39 (29.8%)	92 (70.2%)	0.40
	3 to 5	45 (29.8%)	106 (70.2%)	
	> 5	25 (38.5%)	40 (61.5%)	
Agricultural land ownership ‡	No	33 (33.3%)	66 (66.7%)	0.63
	Yes	76 (30.6%)	172 (69.4%)	
Milk cows or bulls ownership ‡	No	41 (30.2%)	95 (69.8%)	0.68
	Yes	68 (32.2%)	143 (67.8%)	
Primary source of water for drinking ‡	Unimproved*	15 (31.9%)	32 (68.1%)	0.94
	Improved**	94 (31.3%)	206 (68.7%)	
Toilet facility ‡	Unimproved ^ϑ^	38 (32.5%)	79 (67.5%)	0.76
	Improved ^ϑϑ^	71 (30.9%)	159 (69.1%)	

* Unimproved water source - Water from spring, Surface water, rainwater as per the Ethiopia Demographic and Health Survey (EDHS) [19] ** Improved water source- Public tap and dug well [19]

^ϑ^ Unimproved toilet facility- - No facility/ bush/ field [19]

^ϑϑ^ Improved toilet facility- Pit latrine, Ventilated pit latrine, composite toilet [19]

† Marital status and occupation available for *n* = 348

‡ Educational status, family size, Primary source of water for drinking, Milk cows or bulls ownership, primary source of water for drinking, and toilet facility available for *n* = 347

## Discussion

The prevalence of intestinal parasitic infections in late pregnancy is high in rural Amhara where one in three pregnant women harbored parasitic infections in late pregnancy despite prior administration of presumptive mebendazole deworming earlier in the pregnancy. Eventhough, most studies are conducted on the general burden of intestinal parasitic infection during pregnancy regardless of presumptive deworming, our study findings are comparable to the estimated pooled prevalence of IPIs among pregnant women in a systematic review and meta-analysis conducted in Ethiopia [4] and Felege Hiwot Referral Hospital [20]. The prevalence was less than studies conducted in Mecha district, Northwest Ethiopia, Lalo Kile district, Oromia, and Woreilu Health Center, Northeast Ethiopia [11, 21, 22].

Most of the parasites identified in this study population were protozoan infections (Giardia and Amoeba) which are not adequately covered by mebendazole [23]. Secondly, studies in similar settings have shown that the cure rates of hookworm with a single dose of albendazole/ mebendazole treatment were low [24–26]. Certain populations may have a degree of innate resistance to the medications in use. Thus, it is imperative to investigate whether the intestinal parasites in the study area have developed resistance to the widely used anthelmintic, and if alternate regimens should be considered first line. Drug resistance to mebendazole in human hookworms has been reported in Mali [27]. It is also important to study rates of reinfection and good to obtain a baseline stool examination data. Finally, the other factors contributing to to recurrence of IPIs related to poor sanitation and hygiene and low socioeconomic status should be studied.

Thus, this study provides valuable insight into the prevalence of parasitic infections among pregnant women in the current context in Amhara, with implications for altering the current treatment regimens and if supported with further studies to suggest the need for presumptive protozoal treatment. It is critical to understand the type and prevalence of intestinal parasitic infections in the specific setting to better tailor clinical management and recommendations since most anthelmintic and antiparasitic drugs such as metronidazole, praziquantel, pyrantel, and mebendazole/ albendazole are safe to be administered during pregnancy, especially after the first trimester [16]. Identifying the right dose, regime, and type of drug is needed to effectively treat IP during pregnancy and thereby reduce adverse pregnancy outcomes. Thus, future research is needed to inform optimal prophylaxis and treatment regimens in pregnancy.

## Limitations

Stool examination was performed using direct wet mount microscopy, the standard of care at the health center level in Ethiopia [15, 16]. This method has lower sensitivity than other techniques such as the formol-ether concentration technique [18, 28]. However, if this method resulted in the under-detection of infections, then the prevalence of IP infections would be higher than we have reported in the current study. We did not have baseline/pre-treatment stool samples for comparison, and we could not determine the effectiveness of mebendazole or whether individuals were re-infected within a month of testing. The sample size was not adequately powered to look for association with important factors. However, we acknowledge that even though most of the study participants had access to improved water sources, the storage and handling of the water could also serve as one source of infection which we haven’t assessed in our study [29].

## Data Availability

Data will be available upon request.

## Abbreviations

ANC: Antenatal Care
EIMP: Infection Management Package
ENAT: Enhancing nutrition and antenatal infection treatment
GI: Gastrointestinal
